# RETRACTED: Poma et al. In Vitro Modulation of P-Glycoprotein Activity by *Euphorbia intisy* Essential Oil on Acute Myeloid Leukemia Cell Line HL-60R. *Pharmaceuticals* 2021, *14*, 111

**DOI:** 10.3390/ph18101582

**Published:** 2025-10-20

**Authors:** Paola Poma, Manuela Labbozzetta, Aro Vonjy Ramarosandratana, Sergio Rosselli, Marco Tutone, Maurizio Sajeva, Monica Notarbartolo

**Affiliations:** 1 Department of Biological, Chemical and Pharmaceutical Science and Technology (STEBICEF), University of Palermo, 90128 Palermo, Italy; paola.poma@unipa.it (P.P.); manuela.labbozzetta@unipa.it (M.L.); sergio.rosselli@unipa.it (S.R.); marco.tutone@unipa.it (M.T.); maurizio.sajeva@unipa.it (M.S.); 2 Department of Plant Biology and Ecology, University of Antananarivo, P.O. Box 906, Antananarivo 101, Madagascar; arovonjy@yahoo.fr

The journal has retracted the article “In Vitro Modulation of P-Glycoprotein Activity by *Euphorbia intisy* Essential Oil on Acute Myeloid Leukemia Cell Line HL-60R” [1] cited above.

Following publication, concerns were brought to the attention of the Editorial Office regarding an image presented in this publication [1].

Adhering to our complaint procedure, an investigation was conducted by the Editorial Office and Editorial Board that confirmed indications of inappropriate editing within the Western blot presented in Figure 6. While the authors cooperated with the Editorial Office during the investigation, they were unable to satisfactorily explain the irregularities nor provide adequate raw material for the Editorial Board Evaluation. Consequently, the Editorial Board lost confidence in the reliability of the findings and decided to retract this publication [1], as per MDPI’s retraction policy (https://www.mdpi.com/ethics#_bookmark30 accessed on 26 August 2025).

This retraction was approved by the Editor-in-Chief of the journal *Pharmaceuticals.*

The authors did not agree to this retraction.
